# The First Report of *Coxiella burnetii* as a Potential Neglected Pathogen of Acute Hepatitis of Unknown Causes in Egypt

**DOI:** 10.3390/microorganisms10112168

**Published:** 2022-10-31

**Authors:** Mohamed A. El-Mokhtar, Ibrahim M. Sayed, Ayat M. Kamel, Ahmed Atef Mesalam, Elsayed A. Elgohary, Khaled Abo bakr Khalaf, Sara Adel, Azza Abo Elfadl, Walaa A. Khalifa, Haidi Karam-Allah Ramadan

**Affiliations:** 1 Department of Medical Microbiology and Immunology, Faculty of Medicine, Assiut University, Assiut 71515, Egypt; 2 Microbiology and Immunology Department, Faculty of Pharmacy, Assiut University, Assiut 71515, Egypt; 3 Department of Therapeutic Chemistry, Pharmaceutical and Drug Industries Research Institute, National Research Centre (NRC), Cairo 12622, Egypt; 4 Department of Internal Medicine, Faculty of Medicine, Zagazig University, Zagazig 44519, Egypt; 5 Department of Tropical Medicine and Gastroenterology, Faculty of Medicine, Assiut University, Assiut 71515, Egypt; 6 Clinical Pathology Department, Faculty of Medicine Al-Azhar University-Assiut Branch, Assiut 71515, Egypt; 7 Clinical Pathology Department, Faculty of Medicine, Assiut University, Assiut 71515, Egypt; 8 Department of Internal Medicine, Faculty of Medicine, Assiut University, Assiut 71515, Egypt

**Keywords:** hepatitis of unknown causes, Q-fever, *Coxiella burnetii*, coinfection, Egypt

## Abstract

The World Health Organization (WHO) recently alerted the emergence of new pathogens causing acute hepatitis in children across several countries. This new situation directs us to the screening of neglected pathogens that cause acute hepatitis. Q-fever is a zoonotic disease, caused by *Coxiella burnetii*. Although a high seroprevalence of *Coxiella burnetii* was recorded in animals present in Egypt, Q-fever is still a neglected disease, and the diagnosis of Q-fever is not routinely performed in Egyptian hospitals. In this study, we performed a retrospective assessment for *Coxiella burnetii* in cases of hepatitis of unknown causes (HUC) enrolled in Assiut University hospitals, in Egypt. Out of 64 samples of HUC, 54 samples were negative for all hepatitis markers, labeled as acute hepatitis of unknown etiology (AHUE), and 10 samples tested positive for adenovirus and Hepatitis E virus (HEV). Q-fever was detected in 3 out of 54 (5.6%) of AHUE, and one sample was confirmed as coinfection of HEV/Q-fever. Jaundice was the most common clinical symptom developed in the patients. In conclusion, *Coxiella burnetii* was found to be a potential cause of acute hepatitis in HUC. The diagnosis of Q-fever should be considered in acute hepatitis cases in Egyptian hospitals.

## 1. Introduction

The World Health Organization (WHO) has recently reported an outbreak of severe acute hepatitis in children across multiple countries [1,2]. The causative agents were not the known viral hepatitis (Hepatitis viruses A–E), and this alert has underscored the unmet need for screening for emerging or neglected pathogens that are not routinely diagnosed in cases of acute hepatitis of unknown cause.

In Egyptian hospitals, routine screening of hepatitis A virus (HAV), hepatitis B virus HBV, hepatitis C virus (HCV), cytomegalovirus (CMV), and Epstein–Barr virus (EBV) is performed for acute hepatitis (AH) patients. However, a recent study showed many cases of acute hepatitis of unknown etiology (AHUE) in Egypt [3]. These cases only receive supportive treatment, and many of them could progress to fulminant hepatic failure due to missing the actual causative agent and specific therapies. Hepatitis E virus (HEV) is a neglected disease in Egypt and there is no protocol for its screening. Recently, it was reported that 10% of AHUE in Assiut University Hospitals in Upper Egypt were classified as acute HEV infection [3]. Q-fever is another neglected disease in Egypt, and limited data are available on the prevalence of the infection or complications among Egyptians, especially hepatitis patients.

Q-fever is a zoonotic disease, caused by a small pathogen named *Coxiella burnetii*. *C. burnetii* is a Gram-negative bacteria that replicate and survive mainly intracellularly [4]. Animals such as small ruminants, cattle, buffaloes, pets, camels, and wild animals are the main reservoirs of this pathogen [4,5,6,7]. In Egypt, camels and cattle are the main reservoirs of *C. burnetii* [5,8,9]. These animals play an important epidemiological role in the transmission of Q-fever as demonstrated by the increase in the risk of infected or seropositive individuals who were in contact with these animals [10,11]. Infection can be transmitted to humans either through the inhalation of bacteria from infected animals, contact with infected animals, or ingestion of contaminated products such as milk and dairy products [12,13,14]. People who are exposed to animals occupationally, such as farmers and veterinarians, are at higher risk of exposure to Q-fever [13,14]. Q-fever has a wide spectrum of symptoms depending on the immune and health status of patients, and the infection can be acute or chronic [15].

Q-fever is endemic in several countries such as France, Spain, Germany, Italy, Iran, the Middle East, and Taiwan, both in animals and humans [16,17,18,19,20,21]. In Egypt, Q-fever is an enzootic disease, reported in several Governorates and among several animals including cattle, sheep, goats, camels, cats, and rats [14,22,23,24,25,26]. In Assiut Governorate, a recent study reported an unexpectedly high prevalence of *C. burnettii* among livestock (approximately 50%) and among HCV and HBV-infected patients [9]. The previous findings highlight the circulation and endemicity of Q-fever in Egypt. However, the diagnosis of Q-fever is neglected and not routinely performed in Egyptian hospitals.

Herein, we aimed to screen cases of acute hepatitis of unknown causes (HUC) for *C. burnetii*, which could guide clinicians to recommend suitable medications to those patients and reduce the risk of complications.

## 2. Materials and Methods

### 2.1. Patients

The study includes a retrospective analysis of acute hepatitis (AH), acute-on-chronic liver failure (ACLF), and fulminant hepatic failure (FHF) patients admitted to Assiut University Hospital, Al-Rajhi Liver University Hospital, and Assiut Fever Hospital during the period of 2020–2022. Clinical symptoms, laboratory parameters, and abdominal ultrasounds were available for these samples. Plasma samples were analyzed following the protocol of Assiut University Hospital for the diagnosis of AH cases as mentioned previously. Briefly, screening for viral hepatitis (HAV, HBV, and HCV) was performed using ELISA assays for anti-HAV IgM, HBsAg, and anti-HCV antibodies, respectively. The detection of HCV RNA and HBV DNA was performed by qPCR. Screening for anti-CMV IgM, and anti-EBV IgM was performed using commercial ELISA kits (Serion ELISA classic, Germany). For autoimmune hepatitis, total human IgG, anti-smooth muscle antibodies (ASMA), and anti-nuclear antibody IgG were screened. Drug-induced liver injury (DILI) was evaluated depending on the medical history and patient questionnaire as described before [27]. The study was performed in accordance with the Declaration of Helsinki and approved by the Institutional Review Board of the Faculty of Medicine, Assiut University (ID: 17300764). Patients’ consent was not required due to the retrospective analysis.

### 2.2. Definition of the Cases (Figure 1)

(a) Hepatitis of unknown cause (HUC): Any acute hepatitis sample processed by Assiut University Hospital protocol and tested negative for viral hepatitis markers (HAV, HBV, HCV, CMV, and EBV), autoimmune makers, and DILI, which are routinely performed in the hospital.
(b) Acute hepatitis of unknown etiology (AHUE): HUC samples that tested negative for other viral pathogens including adenovirus, COVID-19, Parvovirus, HSV, coxsackie virus, and HEV, which are not routinely performed in the hospital.
(c) Hepatitis caused by less common viral pathogens: HUC samples that tested positive for one or more of the following viral pathogens: Adenovirus, COVID-19, Parvovirus, HSV, coxsackie virus, and HEV.

**Figure 1:**
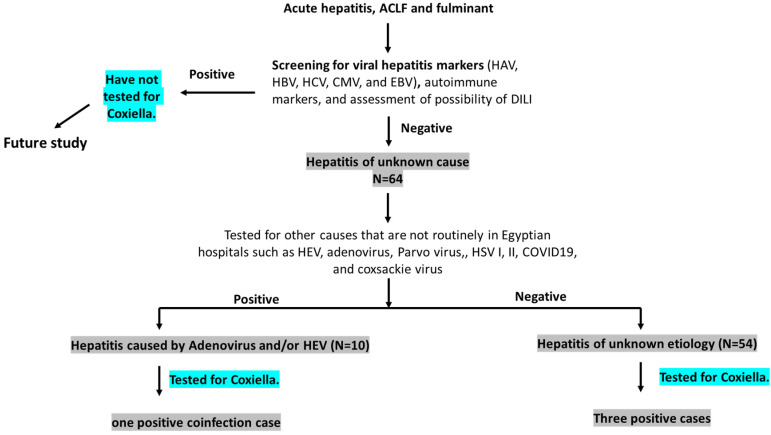
Definition of cases and study design. Sixty-four cases of HUC that tested negative for common viral hepatitis, autoimmune hepatitis markers, and were not drug-induced liver injury (DILI) were screened for less common pathogens. Fifty-four cases were negative for the less common pathogens and labeled as acute hepatitis of unknown etiology (AHUE). Three samples tested positive for Q fever from AHUE. Ten out of 64 samples tested positive for less common pathogens such as HEV and adenovirus, of which one case was diagnosed as HEV/Q-fever coinfection.

### 2.3. Screening of Less Common Viral Pathogens

Screening for anti-HSV IgM, anti-ParvoB19 IgM/IgG, and anti-coxsackie virus IgM/IgG was performed using commercial ELISA kits (Serion ELISA classic, Serion GmbH, Würzburg, Germany). Adenovirus detection was assessed by qPCR using primers and probes targeting a conserved region of the hexon gene as described before [28]. HEV testing was performed via detection of anti-HEV IgM and IgG (the abia, AB Diagnostic Systems GmbH, Berlin, Germany) and/or detection of the HEV ORF2/3 region by qRT-PCR as described previously [29,30].

### 2.4. Molecular Assessment of C. burnetii

DNA was isolated from blood samples using the QIAamp^®^ DNA Mini Kit (Qiagen, Hilden, Germany) according to the manufacturer’s instructions. The detection of C. burnetii DNA was performed by qPCR using primers and probes targeting the IS1111 gene as described previously [31]. The sequence of primers used was forward: 5′-AATTTCATCGTTCCCGGCAG-3′, reverse: 5′-GCCGCGTTTACTAATCCCCA-3′. The TaqMan probe used was 5′-FAM-TGTCGGCGTTTATTGGGTTGGTCCC-BHQ-3′. The PCR reaction was performed using the Applied Biosystems TaqMan universal PCR master mix according to the manufacturer’s instructions. The PCR reactions were run using the Applied Biosystems 7500 Fast (Applied Biosystems, Foster City, CA, USA). 

### 2.5. Statistics

Results were analyzed using GraphPad Prism software 8 (La Jolla, CA, USA). The results are presented as numbers (percentage %), means ± SD, medians with the interquartile range (IQR), or the minimum–maximum range unless otherwise specified. *p*-value was determined by the two-tailed *t*-test or the Mann–Whitney test, as appropriate, for quantitative variables and the Chi square test for categorical variables.

## 3. Results

### 3.1. Molecular Prevalence of Coxiella burnetii Infection in Acute Hepatitis of Unknown (AHUE) Patients

We performed a retrospective analysis on plasma samples diagnosed or labeled as hepatitis of unknown cause (HUC) (N = 64) to assess the prevalence of *C. burnetii*. These samples were collected from acute hepatitis (AH) patients, acute on top of chronic liver failure (ACLF), and fulminant hepatic failure (FHF) patients. Demographic, clinical, and laboratory parameters for these samples are presented in Table 1. Samples that tested negative for common causes of hepatitis such as viral hepatitis (HAV, HBV, HCV, CMV, and EBV), less prevalent causes of viral hepatitis in Egypt including adenovirus, HEV, COVID-19, HSV1, parvovirus, coxsackie virus, autoimmune hepatitis, and DILI, were labeled as AHUE (N = 54) (Figure 1). Jaundice, gastrointestinal symptoms, dark urine, and fever were the common symptoms reported by the patients (Table 1). Parameters of liver function tests (LFTs) were elevated, and abdominal ultrasounds revealed variable patterns among patients. Molecular analysis showed that 3 out of 54 cases (5.6%) were positive for *C. burnetii*.

### 3.2. Clinical Characteristics of Acute Q-Fever Hepatitis among AHUE Patients

All three infected patients were males and were living in rural communities (100%). Jaundice was recorded in all patients, while the other symptoms were variable. Abdominal pain and dark urine were recorded in two out of three patients, and the other symptoms were recorded in one out of three patients. Two patients developed acute hepatitis and the third one had liver cirrhosis and developed ACLF. The LFTs were mildly elevated in the former and highly elevated in the latter (Table 2).

Q-fever-positive patients were older compared to the Q-fever-negative patients, and the predominant gender was male. All other symptoms and laboratory parameters were comparable in both groups (Table 3).

### 3.3. Molecular Prevalence and Clinical Characteristics of Coxiella burnetii Infection in Acute Hepatitis Caused by Less Common Viral Pathogens

Out of 64 samples of AHUC, 10 samples tested positive for other viral pathogens that are not routinely tested in Egyptian hospitals: HEV and adenovirus. Demographic, clinical, and laboratory parameters for these samples are presented in Table 4.

The clinical symptoms were comparable in this group and AHUE, while the liver transaminases (ALT and AST) were elevated in this group compared to AHUE. We screened these samples for *C. burnetti* DNA by qPCR. We found that one sample was positive for the Q-fever marker, and this sample represented a case of coinfection: HEV/Q-fever. This case was a 19-year-old male who complained of fever and vomiting and was hospitalized for 7 days. He took symptomatic treatment and recovered without complications (Table 5). Compared to HEV monoinfection, it seemed that HEV/Q-fever had elevated liver transaminases, lowered the total bilirubin level, and lowered the WBCs count (Table 5).

## 4. Discussion

Recently, the WHO reported new emerging pathogens causing severe hepatitis in children [1]. This alert underscores the importance of screening for emerging pathogens causing acute hepatitis either in children or adults. Although most pathogens causing acute hepatitis are known, the protocol of diagnosis of AH could vary depending on the geographic location, the prevalence of pathogens in the area, and/or the availability of financial and technical supplies required for diagnosis. For example, the diagnosis of HEV and Q-fever is not routinely performed in Egyptian hospitals, though both pathogens are known as causative agents for acute hepatitis. Recently, we conducted a screening of AHUE samples for HEV markers and found that 10% of AHUE in Assiut hospitals were acute hepatitis E cases [3]. However, 90% of AHUE cases in Egypt are still of undetermined origin. Therefore, we aimed to screen these samples for other potential pathogens such as *C. burnetii.*

Several studies confirmed the circulation of *C. burnetii* among ruminants in Egypt suggesting potential zoonotic transmission from these animals to humans [8,9,24,25,26]. The prevalence of infection varies among ruminants distributed in Egyptian governorates depending on the geographic location and assay used. Besides, *C. burnetii* was detected in 33.6% of aborted sheep and 16.3% of aborted goats, indicating a crucial role of the Q-fever in animal abortion [32]. The prevalence of *C. burnetii* can reach 70% in camels [5,8]. In addition, *C. burnetii* was detected in rats (6.7%) and parturient cats (7.5%), but not in dogs, suggesting that these animals could be potential reservoirs for C. *burnetii* in Egypt [22,23]. The seroprevalence of *C. burnetii* IgG antibodies among sheep and goat breeders (25.71%) was comparable to the percentages of infection in the animals [24]. In Iran, the herd prevalence of C. *burnetii* in small ruminants (sheep and goats) was over 90% and 41% in herd cattle [21]. In Assiut Governorate, a recent report recorded a high prevalence of anti- *C. burnetii* IgG antibodies among livestock (45–60% depending on the assay) and humans (53–55%) [9]. The screened human samples were selected randomly and were either from patients with known hepatitis (HBV or HCV) or patients from higher-risk groups (kidney dialysis patients, fever of unknown cause, or flu patients). To our knowledge, this is the first study to assess the molecular prevalence and clinical characteristics of acute Q-fever hepatitis in HUC in Egypt.

In the current study, 4 (6.25%) out of 64 cases of HUC were Q-fever. The infected patients were males (100%) with a mean age of 42.75 ± 18.45 years. Similarly, Melenotte et al. assessed the features of Q-fever patients treated at the French National Reference Center for Q fever over 26 years (1991–2016) and reported that 68% of patients were male, with a mean of age of 51 years [15]. Interestingly, a study executed on mice revealed that sex plays a crucial role in the pathogenesis of *C. burnetii* [33]. Higher bacterial load and granuloma numbers were recorded in male mice compared to females, and the previous tissue damage effect was protected by 17β-Estradiol, suggesting that estrogen reduces the inflammatory responses and destruction mediated by *C. burnetii* [33]. The clinical symptoms of Q-fever patients in this study are variable. Jaundice developed in 75% of the patients, and fever, vomiting, dark urine, and abdominal pain were recorded in 50% of patients while diarrhea, ascites, and encephalopathy were detected in 25% of patients. Likewise, analysis of the clinical presentations of acute Q-fever patients (N = 1806) revealed that hepatitis was the most common feature (46.3%), followed by pneumonia (26.6%), while flu-like syndrome or isolated fever represented 19.3% of the cases [15]. Moreover, several case reports documented the development of cholestatic jaundice or hyperbilirubinemia with Q-fever [34,35]. Contrary to our findings, other studies showed that fever is the common feature of acute Q-fever, while jaundice is rare [36,37]. Other reported symptoms in our study, such as abdominal pain, diarrhea, and vomiting, were also reported by others [36,37]. Acute Q-fever has a wide and variable range of manifestations, which could be affected by geographic location, age, gender, immune and health status, pregnancy, time of hospitalization and sample collection, etc.

Mild liver transaminases were reported in the three cases infected with *C. burnetii*, while liver transaminases were higher in the fourth case, likely due to coinfection of HEV and Q-fever. To our knowledge, this is the first report that describes a coinfection between HEV and Q-fever. It seems that the HEV/*C. burnetii* coinfection case had higher liver transaminases and lower bilirubin levels compared to mono-infection of HEV or *C. burnetii*. However, due to the small sample size, future studies are needed to confirm these points. Interestingly, our recent studies reported the circulation of HEV among ruminants and their dairy products (milk) in the Assiut Governorate [38,39,40]. The previous findings suggest that ingestion of ruminant products could be the common method of transmission of HEV and Q-fever, especially since all Q-fever patients in this study are from the rural community. Though data on the coinfection of *C. burnetii* with other pathogens are limited, coinfection of Q-fever with other hepatotropic agents such as HBV, HCV, and CMV was documented [41,42]. However, the outcomes were variable. Lai and colleagues reported that the clinical symptoms of Q-fever were not affected by coinfection with HBV or HCV and the bacteria did not affect the viral replication [41]. Meanwhile, coinfection of CMV/Q-fever was associated with fulminant hepatic and renal failures [42]. Future studies need to assess the impact of other *Coxiella* coinfection.

There were no differences in the clinical symptoms between Q-fever and AHUE, suggesting that the laboratory identification of *C. burnetii* is the main way to a diagnosis of Q-fever. Previously, we had reported that liver transaminases (ALT and AST) could be valuable markers to distinguish between suspected and non-suspected HEV in DILI cases [27]. However, this was not the case in Q-fever. Likewise, other studies showed that it was difficult to discriminate between Q-fever and other causes of hepatitis based on clinical symptoms [41,43].

Liver biopsies of Q-fever patients are characterized by granuloma hepatitis, while granuloma is identified by the fibrin ring [44].

This study had several limitations. First, the sample size was small, and the number of positive Q fever cases was also small. The small sample size is due to our target samples (HUC and AHUE) which should be negative for all known hepatitis causes. The limited samples could explain the insignificant statistics results comparing Q-fever-positive AHUE and Q-negative AHUE. Secondly, the study is retrospective, therefore monitoring the patients and clinical symptoms over time was not available. We could not proceed if chronic hepatitis or further complications developed after hospitalization. Future studies including larger sample sizes and prospective in nature should be performed. Third, we could not assess the liver pathologies in those patients due to the absence of liver biopsies. Q-fever-associated liver pathology is characterized by doughnut lesions, which are granulomatous tissues with clear central space and fibrinoid rings [45]. Fourth, as shown in Figure 1, we only screened AHUE for *C. burnetii*; however, hepatitis samples of known etiologies (such as HBV, HCV, CMV, EBV, and autoimmune hepatitis) were not included in the study. Therefore, we believe that the actual prevalence of Q-fever in hepatitis patients in Egypt is still underestimated.

Despite the previous limitations, our study has several strengths; (1) It is the first study that assesses the molecular prevalence and describes the clinical characteristics of Q-fever in AHUC in Egypt. (2) It is the first study that shows coinfection of HEV/Q-fever and the possibility of one mode of transmission. (3) Our study underscores the importance of the inclusion of Q-fever diagnosis in Egyptian hospitals, especially in cases of HUC. (4) Since several animals are considered reservoirs for *C. burnetii* in Egypt, Q-fever should not be neglected or remain an undetermined disease and it is recommended that Q-fever be considered in all hepatitis cases, not only AHUE.

## 5. Conclusions

*C. burnetii* was detected in HUC in Egyptian hospitals. Coinfection of *C. burnetii* and HEV was reported. The clinical symptoms of Q-fever are comparable to AHUE. It is recommended to perform laboratory diagnosis of *C. burnetii* in hepatitis cases enrolled in Egyptian hospitals.

## Figures and Tables

**Table 1:** Demographic, clinical, and laboratory parameters of acute hepatitis of unknown etiology (AHUE).

Parameter	Acute Hepatitis of Unknown Etiology (AHUE) (N = 54) (n, %)
Age (years) *	38 (27–45)
Gender	
Male	35 (64.8%)
Female	19 (35.2%)
Clinical symptoms	
Jaundice	51 (94.4%)
Fever	20 (37.03%)
Vomiting	32 (59.26%)
Abdominal pain	32 (59.26%)
Diarrhea	21 (38.9%)
Dark urine	25 (46.3%)
Ascites ^$^	4 (7.4%)
Encephalopathy	6 (11.1%)
Liver functions (LF) *	
ALT (IU/L)	106 (72–303)
AST (IU/L)	107 (67–347)
Total bilirubin (μmol/L)	230 (142–272)
Direct bilirubin (μmol/L)	153 (69–198)
Albumin (g/L)	34 (29–38)
Abdominal Ultrasound	
Normal	39 (72.2%)
Fatty Liver	5 (9.26%)
Hepatomegaly	3 (5.56%)
Hepatosplenomegaly	3(5.56%)
Cirrhosis	4 (7.4%)
Course of hepatitis	
Acute hepatitis (AH)	43 (79.63%)
Acute on top of chronic liver failure (ACLF)	4 (7.4%)
Fulminant hepatitis (FH)	7 (12.96)

* Values are represented as medians and interquartile ranges (IQR). ^$^ Ascites: Mild form in all cases. ALT: Alanine aminotransferase (normal: 0–45 IU/L); AST: Aspartate aminotransferase (normal: 0–34 IU/L).

**Table 2:** Demographic, clinical, and laboratory criteria of acute Q-fever patients.

	Patient #1	Patient #2	Patient #3
Age	43	45	64
Sex	Male	Male	Male
Clinical symptoms			
Jaundice	Yes	Yes	Yes
Fever	No	Yes	No
Vomiting	No	Yes	No
Abdominal pain	Yes	Yes	No
Diarrhea	No	Yes	No
Dark urine	Yes	No	Yes
Ascites	No	No	Yes
Encephalopathy	No	No	Yes
Liver functions (LF)			
ALT (IU/L)	92	67	1354
AST (IU/L)	61	88	630
Total bilirubin (μmol/L)	263	235	233
Direct bilirubin (μmol/L)	155	156	181
Albumin (g/L)	31	33	24
Abdominal Ultrasound	Normal	Normal	Cirrhosis
Course of hepatitis	Acute hepatitis	Acute hepatitis	ACLF
Blood pictures			
WBCs (×1000/mm^3^)	7.5	7.6	6.3
Platelets (×1000/mm^3^)	265	246	170
International normalized ratio (INR)	1.1	1.01	1.9
Risk factor			
Rural/urban	rural	Rural	rural

**Table 3:** Comparison between Q-fever-positive and Q-fever-negative AHUE patients.

	Q-Fever–Positive N = 3	Q-Fever–Negative N = 51
Age mean ± SD	50.67 ± 11.6	37.8 ± 12.73
Sex	Male: 3/3 (100%) Female: 0/3 (0%)	Male: 32/51 (62.7%) Female: 19/51 (37.3%)
Clinical symptoms		
Jaundice	3/3 (100%)	48/51 (94.1%)
Fever	1/3 (33.3%)	19/51(37.25%)
Vomiting	1/3 (33.3%)	31/51 (60.78%)
Abdominal pain	2/3 (66.7%)	30/51 (58.8%)
Diarrhea	1/3 (33.3%)	20/51 (39.2%)
Dark urine	2/3 (66.7%)	23/51 (45.1%)
Ascites	1/3 (33.3%)	3/51 (5.9%)
Encephalopathy	1/3 (33.3%)	5/51 (9.8%)
Liver functions (LF) *		
ALT (IU/L)	92	112
AST (IU/L)	88	108
Total bilirubin (μmol/L)	235	220.6
Direct bilirubin (μmol/L)	156	150.8
Albumin (g/L)	31	34
Blood pictures		
WBCs (×1000/mm^3^)	7.13 ± 0.72	7.54 ± 2.8
Platelets (×1000/mm^3^)	227 ± 50.27	214 ± 80.22
International normalized ratio (INR)	1.34 ± 0.49	1.3 ± 0.64

* expressed as Median. There were no statistically significant differences between the two groups in all parameters. *p*-values were more than 0.05.

**Table 4:** Demographic, clinical, and laboratory parameters of acute hepatitis caused by less common viral pathogens.

Parameter	Acute Hepatitis Caused by Less Common Viral Pathogens (N = 10) (n, %)
Age (years) *	33 (21–39)
Gender	
Male	6 (60%)
Female	4 (40%)
Clinical symptoms	
Jaundice	9 (90%)
Fever	3 (30%)
Vomiting	2 (20 %)
Abdominal pain	3 (30%)
Diarrhea	0 (0%)
Dark urine	4 (40%)
Ascites	4 (40 %)
Encephalopathy	3 (30%)
Liver functions (LF) *	
ALT (IU/L)	519 (250–994)
AST (IU/L)	428 (236–817)
Total bilirubin (μmol/L)	164 (86–287)
Direct bilirubin (μmol/L)	132 (56–215)
Albumin (g/L)	25 (21–33)
Abdominal Ultrasound	
Normal	5 (50%)
Hepatomegaly	1 (10%)
Cirrhosis	4 (40%)

* Values are represented as medians and interquartile ranges (IQR).

**Table 5:** Characteristics of acute hepatitis caused by HEV and coinfection HEV/Coxiella.

	HEV Infection N = 4	HEV/Coxiella N = 1
Age (minimum–maximum)	21–37	19
Sex	Male: 1/4 (25%) Female: 3/4 (75%)	Male
Clinical symptoms		
Jaundice	4/4 (100%)	No
Fever	1/4 (25%)	Yes
Vomiting	1/4 (25%)	Yes
Abdominal pain	1/4 (66.7%)	No
Diarrhea	0/4 (0%)	No
Dark urine	1/4 (25%)	No
Ascites	2/4 (50%)	No
Encephalopathy	2/4 (50%)	No
Liver functions (LF)		
ALT (IU/L)	272–659	1544
AST (IU/L)	264–1141	410
Total bilirubin (μmol/L)	147–414	80
Direct bilirubin (μmol/L)	118–325	60
Albumin (g/L)	20–28	40
Blood pictures		
WBCs (×1000/mm^3^)	4.1–12.7	3
Platelets (×1000/mm^3^)	92–264	144
International normalized ratio (INR)	1–2.5	1.1

Results expressed as minimum–maximum.

## Data Availability

All the data regarding this study are present in the manuscript. For further inquiries, please contact the first and corresponding authors.
